# PI3K pathway protein analyses in metastatic breast cancer patients receiving standard everolimus and exemestane

**DOI:** 10.1007/s00432-020-03291-x

**Published:** 2020-06-21

**Authors:** Dinja T. Kruger, Mark Opdam, Vincent van der Noort, Joyce Sanders, Michiel Nieuwenhuis, Bart de Valk, Karin J. Beelen, Sabine C. Linn, Epie Boven

**Affiliations:** 1 Department of Medical Oncology, Amsterdam UMC, Vrije Universiteit Amsterdam/Cancer Center Amsterdam, De Boelelaan 1117, 1081 HV Amsterdam, The Netherlands; 2 Division of Molecular Pathology, The Netherlands Cancer Institute, Plesmanlaan 121, 1066 CX Amsterdam, The Netherlands; 3 Division of Biometrics, The Netherlands Cancer Institute, Plesmanlaan 121, 1066 CX Amsterdam, The Netherlands; 4 Department of Pathology, The Netherlands Cancer Institute, Plesmanlaan 121, 1066 CX Amsterdam, The Netherlands; 5 Department of Internal Medicine - Oncology, Spaarne Gasthuis, Spaarnepoort 1, 2134 TM Hoofddorp, The Netherlands; 6 Department of Internal Medicine - Oncology Centre, Reinier de Graaf Gasthuis, Reinier de Graafweg 5, 2625 AD Delft, The Netherlands; 7 Department of Medical Oncology, The Netherlands Cancer Institute, Plesmanlaan 121, 1066 CX Amsterdam, The Netherlands; 8 Department of Pathology, University Medical Centre Utrecht, and Utrecht University, Heidelberglaan 100, 3584 CX Utrecht, The Netherlands

**Keywords:** Breast cancer, Everolimus, Exemestane, Phosphatidylinositol-3-kinase, Mitogen-activated protein kinase, p-4EBP1

## Abstract

**Purpose:**

Everolimus plus exemestane (EVE/EXE) is a registered treatment option for ER-positive, HER2-negative (ER +/HER2-) metastatic breast cancer (MBC), but resistance mechanisms limit efficacy. We aimed to find markers that might help select patients with a higher chance on benefit from EVE/EXE.

**Methods:**

Immunohistochemistry (IHC) of PTEN, p-AKT(Thr308), p-AKT(Ser473), p-4EBP1, p-p70S6K, p-S6RP(Ser240/244), p-ERK1/2 and p-S6RP (Ser235/236) was performed on primary tumour tissue and on biopsies immediately taken from ER +/HER2- MBC patients before the start of standard EVE/EXE (Eudract 2013-004120-11). Unsupervised hierarchical clustering was executed to create heatmaps to distinguish subgroups of preferentially activated and less-activated PI3K/MAPK proteins. Uni- and multivariate Cox models were used for associations with PFS.

**Results:**

Primary tumour tissue from 145 patients was retrieved. Median PFS was 5.4 months. Patients without (neo)adjuvant therapy (*p* = 0.03) or bone only disease (*p* = 0.04) had longer PFS on EVE/EXE. In primary tumours, neither single proteins nor PI3K/MAPK-associated heatmap subgroups were significantly associated with PFS. In 21 patients a non-osseous biopsy obtained before dosing was useful for continuous scoring, which demonstrated upregulation of several proteins as compared to readings in corresponding primary tumour tissues. These comparisons revealed that increased expression of p-4EBP1 was significantly associated with worse PFS (multivariate HR 3.69, *p* = 0.05).

**Conclusions:**

IHC of single proteins or heatmap subgroups of the differentially activated PI3K/MAPK pathways was not able to discriminate patients on EVE/EXE with poor or better PFS. Upregulation of p-4EBP1 in pre-treatment biopsies as compared to levels in primary tumours pointed towards shorter PFS.

**Electronic supplementary material:**

The online version of this article (10.1007/s00432-020-03291-x) contains supplementary material, which is available to authorized users.

## Introduction

In patients with advanced oestrogen receptor (ER)-positive metastatic breast cancer (MBC) without rapidly progressing visceral metastases, endocrine therapy is first choice of treatment (Partridge et al. 2014). Unfortunately, disease progression will occur in all MBC patients due to de novo or acquired endocrine resistance. Activation of the phosphatidylinositol 3-kinase (PI3K) pathway (Miller et al. 2011a; Tryfonidis et al. 2016), the most frequently altered signalling route in breast cancer (Cancer Genome Atlas 2012), is one of the identified mechanisms that may contribute to endocrine resistance. An activated PI3K pathway can promote RNA translation, proliferation, cell growth and survival (Ciruelos Gil 2014), processes important for tumour cell function. This pathway can interact with ER signalling through cross-talks (Ciruelos Gil 2014; Osborne and Schiff 2011), which may diminish endocrine sensitivity. Endocrine sensitivity may be further affected by interaction of the PI3K pathway with the mitogen-activated protein kinase (MAPK) pathway (Guegan et al. 2014; Saini et al. 2013; Tolcher et al. 2018).

Everolimus, a rapamycin analogue, inhibits the mammalian target of rapamycin-containing complex 1 (mTORC1), which is a key component in the PI3K pathway. Blocking mTORC1 may prevent tumour growth caused by activation of this pathway. The randomised phase 3 BOLERO-2 study has demonstrated in patients with MBC refractory to a non-steroidal aromatase inhibitor (NSAI) that everolimus combined with exemestane (EVE/EXE) resulted in improved progression-free survival (PFS) compared to that obtained with exemestane plus placebo (7.8 vs 3.2 months, respectively, by investigator review) (Yardley et al. 2013). A longer PFS than would have been expected from exemestane alone was demonstrated in various open-label trials on EVE/EXE for treatment of NSAI-refractory MBC. However, this combination is not effective in all patients at the cost of considerable adverse events (Rugo et al. 2014).

We here present the results of immunohistochemistry (IHC) analyses in tumour tissue on PI3K/MAPK pathway activation from 145 MBC patients that received standard EVE/EXE in an attempt to find a potential marker helpful to select patients for this type of treatment. We analysed five phosphorylated proteins in the PI3K pathway (Miller et al. 2011b), p-AKT(Thr308), p-AKT(Ser473), p-4EBP1, p-p70S6K, p-S6RP(Ser240/244), as well as PTEN in primary tumour tissue and in a number of tumour biopsies immediately taken before drug dosing and linked these results with PFS. We also hypothesised that activation of the MAPK pathway might affect treatment outcome due to cross-talk with the PI3K pathway (Yi and Ma 2017). Phosphorylation of S6RP at all phosphosites occurs by p-p70S6K, but p-S6RP at Ser235/236 may represent an activated MAPK pathway (Meyuhas 2015; Roux et al. 2007). Therefore, p-ERK1/2 (Saini et al. 2013) and p-S6RP(Ser235/236) were analysed whether MAPK activation might be related with PFS in the same patients.

## Materials and methods

### Study design

The Everolimus Biomarker Study was an open-label, single arm, multicentre study (EudraCT number 2013-004120-11) designed to gain more insight into tumour characteristics that help select which patients would have a high chance on a long PFS while using EVE/EXE. Eligibility criteria can be found in the Supplements. Patients were included between March 2014 and February 2017 by 28 hospitals in the Netherlands (Table S1).

Patients received everolimus 10 mg and exemestane 25 mg orally per day in cycles of 28 days. A starting dose of 5 mg daily for everolimus was allowed to prevent stomatitis in frail patients, but in the absence of symptoms this dose had to be increased to 10 mg in the next 2 weeks. Dose interruptions or modifications because of adverse events suspected to be related to everolimus were carried out according to protocol guidelines. Clinical examination of patients was carried out every 28 days. Tumour measurements by radiographic assessments were performed preferably every 12 to determine treatment efficacy.

### Immunohistochemistry and scoring

Formalin-fixed paraffin-embedded (FFPE) blocks from the primary tumour of the participating patients were collected. Tissue microarrays (TMAs) were constructed using three 0.6 mm cores taken from the blocks. In those cases, where TMAs could not be made, whole slides of the blocks were prepared for staining procedures. When a patient gave informed consent for a biopsy, preferably 14 Gy, of an accessible non-osseous metastatic site, this procedure was carried out before the start of treatment with EVE/EXE (pre-treatment biopsy).

**Fig. 1:**
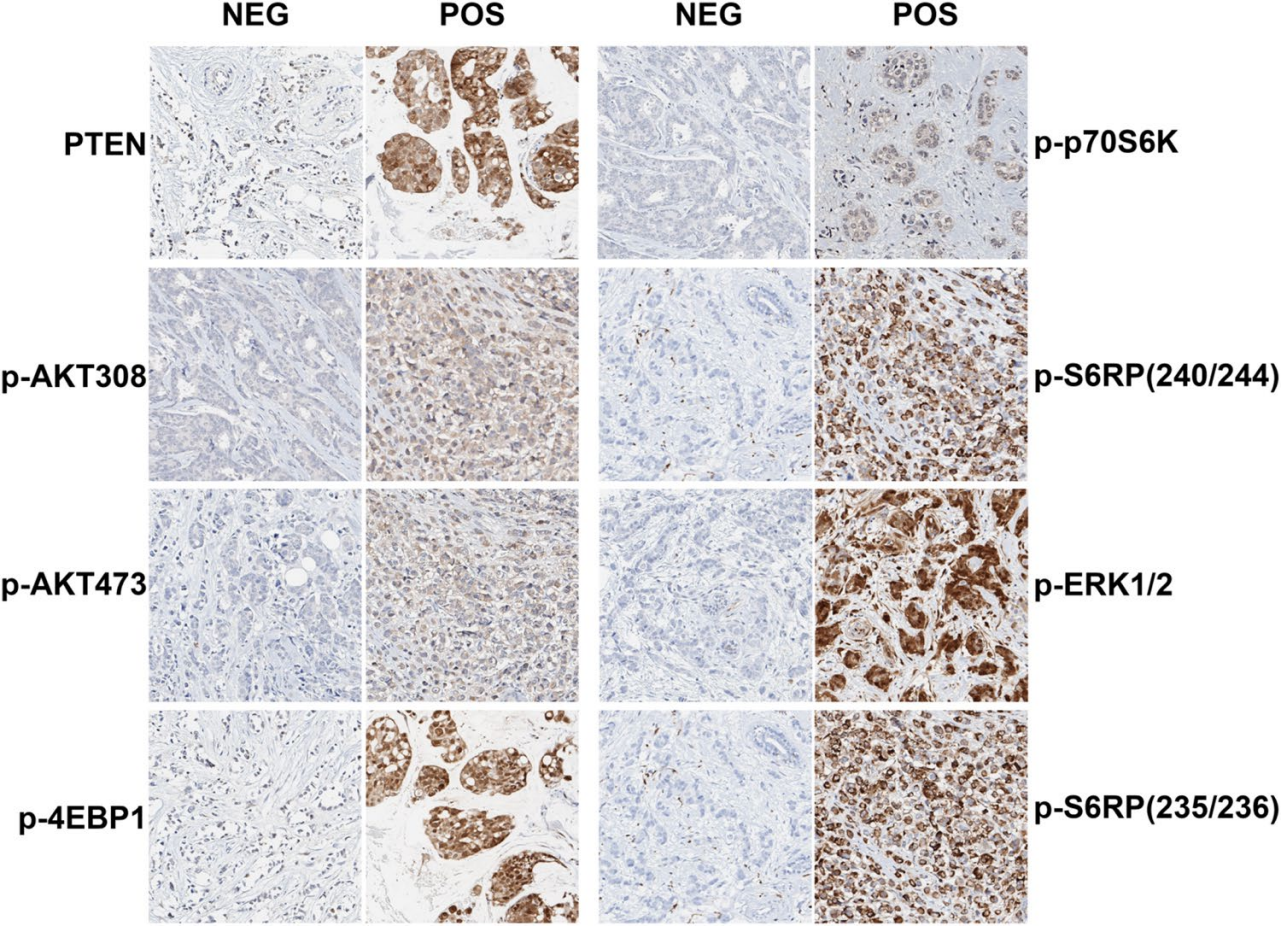
Representative images of IHC staining of PTEN, p-AKT(Thr308), p-AKT(Ser473), p-4EBP1, p-p70S6K, p-ERK1/2, p-S6RP(Ser235/236) and p-S6RP(Ser240/244). NEG: example of negative staining. POS: example of positive staining

To study the effect of activation of the PI3K pathway on EVE/EXE PFS, samples were stained for six proteins associated with this pathway [PTEN, p-AKT(Thr308), p-AKT(Ser473), p-4EBP1(Ser65), p-p70S6K(Thr389) and p-S6RP(Ser240/244)]. MAPK pathway activation was analysed by staining for p-ERK1/2(Thr202/Tyr204) and p-S6RP(Ser235/236). The manufacturer provided standard staining protocols and several dilutions were tested. Variation of the staining for all antibodies was further tested on cancer cell lines or (tumour) tissues. A pathologist assessed and approved all tests before implementation of the antibodies and staining protocols in our institution. After implementation, control cell lines or (tumour) tissue were added in each standardised staining run to check the quality of the staining. Methodology for staining of PTEN, p-AKT(Thr308), p-AKT(Ser473), p-4EBP1, p-p70S6K, p-ERK1/2 and p-S6RP(Ser235/236) has previously been published (Beelen et al. 2014; Kruger et al. 2018). Staining and validation using a similar methodology was carried out for p-S6RP(Ser240/244). Representative IHC images are shown in Fig. 1. One stained TMA was assessed by a second blinded observer to determine the interobserver variability. The scoring results of both the percentage of stained tumour cells (0–100%) and intensity of the staining (0–3) were analysed as binary factor using the median as cutoff to calculate the interobserver variability expressed as kappa coefficient (McHugh 2012). The scoring result with the best kappa coefficient (percentage vs. intensity) was selected (Table S2). For further analyses we used the scores generated by one observer (MO).

### Statistics

PFS was calculated as the time from the start of treatment until radiological progression of disease, clear progression by physical examination or death by any cause. If treatment was discontinued without evidence of progression, patients were censored at the time-to-treatment switch. Patients who were still on everolimus or exemestane at the data lock (1 March 2018) were censored at the last confirmed date of treatment exposure. Patients that stopped treatment with both EVE/EXE within the first month were excluded from the analysis.

Binary scores from all eight proteins stained in primary tumour tissue (cutoffs shown in Table S2) were analysed for their association with PFS. Unsupervised hierarchical clustering was performed to generate a heatmap of the continuous scorings of proteins related with an activated PI3K pathway. Methodology of the heatmap procedure has recently been published (Kruger et al. 2018). Heatmap subgroups were formed representing a preferentially activated (*A*) or less-activated (*N*) PI3K pathway. The effect of MAPK activation on PFS with EVE/EXE was evaluated by combining p-ERK1/2 and p-S6RP(Ser235/236). When p-ERK1/2 was positive in combination with high levels of p-S6RP(Ser235/236), the pathway was considered activated. In an additional experiment we performed unsupervised hierarchical clustering with the continuous scores of the PI3K/MAPK pathway proteins as described before (Kruger et al. 2018) and compared PFS between subgroups with and without preferentially activated pathways.

In pre-treatment biopsies, analyses were performed with the binary scores for PTEN, p-AKT(Thr308), p-AKT(Ser473), p-4EBP1, p-p70S6K, p-ERK1/2 and p-S6RP(Ser235/236) for an association with PFS. The continuous staining score of each individual protein was used to compare readings in the pre-treatment biopsies with those in the corresponding primary tumours. When a score in a pre-treatment biopsy was higher than that in the primary tumour, it was classified as upregulated. Since even a partial inactivation of PTEN is associated with more activation of the PI3K pathway (Alvarez-Garcia et al. 2019), we performed an additional analysis for possible downregulation of PTEN in pre-treatment biopsies.

PFS curves were generated with the Kaplan–Meier method. Cox logistic regression with uni- and multivariate analyses was used to calculate hazard ratios, 95% confidence intervals (95% CI) and *p*-values. Clinicopathological characteristics considered for inclusion as covariates in the multivariate analyses were determined by backwards elimination and were: age (< 65 vs ≥ 65), ECOG status screenings visit (0 vs 1–2), disease-free interval (DFI) (< 12 months vs ≥ 12 months), progesterone receptor status primary tumour (positive vs negative), tumour stage (T1–2 vs T3–4), (neo)adjuvant therapy (which could be hormonal therapy, chemotherapy or both) (yes vs no), bone only disease (yes vs no) and previous palliative chemotherapy (yes vs no). The elimination began with a multivariate Cox model with all characteristics after which the least significant factor was eliminated to generate the next Cox model. This elimination of the least significant factor continued until all characteristics left in the multivariate Cox model had *p*-values of ≤ 0.1. The relative quality of all generated Cox models was checked by the Akaike’s information criterion. Using this method, we determined that the following characteristics had to be included in the multivariate analyses to investigate the effect of the various proteins on PFS: progesterone receptor status of the primary tumour, (neo)adjuvant therapy, bone only disease and palliative chemotherapy.

To test whether PI3K/MAPK protein expression levels in primary tumour tissues or pre-treatment biopsies were associated with PFS in patients receiving EVE/EXE, uni- and multivariate Cox proportional hazard regression analyses were performed including the characteristics mentioned in the previous paragraph.

The study complied with reporting recommendations for tumour marker prognostic studies (REMARK) criteria. Statistical analyses were generated with SPSS 22.0 (IBM SPSS, Illinois, USA) and R for statistics (Windows version 3.3.1). All *p* values were two-sided and significance was defined at ≤ 0.05.

## Results

### Association of PFS with clinicopathological characteristics and protein expression in primary tumours

178 patients signed informed consent for the Everolimus Biomarker Study of which two patients were excluded who did not meet the in- and exclusion criteria and one who never started treatment (Fig. S1). In this group of 175 patients, median PFS was 5.3 months (95% CI: 4.77–5.87) ranging from 0.46 to > 36.8 months. Primary tumour tissue could be retrieved from 145 patients. Median PFS in these patients was 5.4 months (95% CI 4.86–5.91) ranging from 0.46 to > 36.8 months. Patients who were excluded from the IHC analyses had no primary tumour tissue available (*N* = 26) or discontinued treatment within cycle 1 due to toxicity (*N* = 4). There were no differences in clinicopathological characteristics between the total study population and the group included in the current study (Table 1).

**Table 1:** Clinicopathological characteristics of the total study, the primary tumour tissue population and patients with a pre-treatment biopsy

		Total study population	Patients with data primary tumour	Patients with data extra biopsy
Number of patients		*n *= 175	*n *= 145	*n *= 21
Age	Median (range)	63 (34 - 90)	63 (34 - 90)	59 (45–78)
Disease-free interval^a^	Median (range)	72 (0 - 304)	76 (0 - 304)	78 (0–255)
	<12 months, *n* (%)	37 (21)	31 (21)	3 (14)
	12 - 24 months, *n* (%)	9 (5)	8 (6)	1 (5)
	> 24 months, *n* (%)	129 (74)	106 (73)	17 (81)
(Neo)adjuvant therapy, *n* (%)	No (neo)adjuvant therapy, *n* (%)	76 (43)	64 (44)	6 (29)
	Only chemotherapy, *n* (%)	9 (5)	8 (6)	1 (5)
	Only endocrine therapy, *n* (%)	17 (10)	13 (9)	2 (9)
	Both, *n* (%)	73 (42)	60 (41)	12 (57)
Progesterone receptor status primary tumour, *n* (%)	Positive (≥ 10%), *n* (%)	128 (73)	107 (74)	17 (81)
	Negative (< 10%), *n* (%)	33 (19)	31 (21)	2 (9)
	Missing, *n* (%)	14 (8)	7 (5)	2 (9)
Metastatic sites, *n* (%)	Bone, *n* (%)	156 (89)	130 (90)	18 (86)
	Brain, *n* (%)	5 (3)	4 (3)	0
	Breast, *n* (%)	14 (8)	11 (8)	3 (14)
	Liver, *n* (%)	76 (43)	57 (39)	12 (57)
	Lung, *n* (%)	59 (34)	42 (29)	8 (38)
	Lymph nodes, *n* (%)	63 (36)	53 (37)	12 (57)
	Skin, *n* (%)	7 (4)	6 (4)	3 (14)
	Other, *n* (%)	50 (29)	41 (28)	6 (29)
Number of metastatic sites, *n* (%)	1, *n* (%)	31 (18)	27 (19)	
	2, *n* (%)	62 (35)	50 (34)	7 (33)
	≥ 3, *n* (%)	82 (47)	68 (47)	14 (67)
ECOG performance status, *n* (%)	0, *n* (%)	68 (39)	58 (40)	9 (43)
	1, *n* (%)	96 (55)	80 (55)	11 (52)
	2, *n* (%)	11 (6)	7 (5)	1 (5)
Number of lines of endocrine therapy in metastatic setting, n (%)^b^	0, *n* (%)	17 (10)	15 (10)	6 (29)
	1, *n* (%)	57 (33)	47 (33)	5 (24)
	2, *n* (%)	67 (38)	55 (38)	7 (33)
	≥ 3, *n* (%)	34 (19)	28 (19)	3 (14)
Number of lines of chemotherapy in metastatic setting, *n* (%)	0, *n* (%)	125 (71)	103 (71)	15 (71)
	1, *n* (%)	24 (14)	21 (15)	3 (14)
	2, *n* (%)	15 (9)	12 (8)	1 (5)
	≥ 3, *n* (%)	11 (6)	9 (6)	2 (9)

^a^Disease-free interval (DFI) is defined as the time from diagnosis of primary breast cancer to first relapse in months. From the patients with < 12 months DFI, all but one had stage IV disease at first presentation

^b^Different aromatase inhibitors are counted as separate lines

In univariate analyses, patients who did not receive (neo)adjuvant therapy (*p* = 0.03) or with bone only disease (*p* = 0.04) had significantly better PFS on EVE/EXE (Table 2). In the multivariate analyses, PFS in patients who had not received (neo)adjuvant therapy remained significantly improved (*p* = 0.01). Also, PFS in patients without palliative chemotherapy was significantly better than that in those on previous palliative chemotherapy (*p* = 0.05) (Table 2). None of the single PI3K/MAPK proteins were associated with PFS benefit from EVE/EXE in the univariate or the multivariate analyses (Table 2). The Kaplan–Meier curves of the PFS for the various proteins split by binary scores are visualised in Figure S2. Graphically, the curves of p-4EBP1 ≤ 50% and > 50% seem to demonstrate a difference in PFS, although not significant in both the univariate (HR 1.48, *p* = 0.06) and multivariate analysis (HR 1.4, *p* = 0.11) (Table 2).

**Table 2:** Univariate and multivariate Cox analyses of progression-free survival in relation with clinicopathological characteristics or PI3K/MAPK pathway proteins in primary tumour tissues

		Progression-free survival						
		*N*	Univariate analyses			Multivariate analyses		
			HR	95% CI	*p* value	HR	95% CI	*p*-value
Age	< 65 years	80	1					
	≥ 65 years	65	0.93	0.66–1.31	0.69			
ECOG status screenings visit	0	58	1					
	1–2	87	1.25	0.88–1.76	0.22			
Disease-free interval	< 12 months	31	1					
	≥ 12 months	114	1.06	0.70–1.62	0.77			
Tumour stage	T 1–2	94	1					
	T 3–4	33	1.20	0.79–1.84	0.39			
Progesterone receptor status	Negative	31	1			1		
of the primary tumour	Positive	107	0.80	0.52–1.22	0.29	0.70	0.45–1.07	0.10
(Neo)adjuvant therapy^a^	No	64	1			1		
	Yes	81	1.47	1.04–2.09	**0.03**	1.62	1.12–2.34	**0.01**
Bone only disease	No	120	1			1		
	Yes	25	0.61	0.38–0.97	**0.04**	0.66	0.41–1.06	0.09
Palliative chemotherapy	No	103	1			1		
	Yes	42	1.42	0.98–2.05	0.07	1.48	1.00–2.19	**0.05**
PTEN	0	13	1			1		
	1–3	129	1.23	0.68–2.24	0.50	0.98	0.52–1.83	0.94
p-AKT (Thr308)	0	9	1			1		
	1–3	135	0.92	0.47–1.82	0.81	0.91	0.42–1.99	0.81
p-AKT (Ser473)	0–1	99	1			1		
	2–3	46	1.22	0.84–1.76	0.30	1.08	0.74–1.58	0.70
p-4EBP1 (Ser65)	0–50%	36	1			1		
	51–100%	109	1.48	0.99–2.21	0.06	1.43	0.93–2.21	0.11
p-p70S6K (Thr389)	0	40	1			1		
	1–3	105	1.03	0.70–1.52	0.78	1.04	0.69–1.56	0.87
p-S6RP (Ser240/244)	0–10%	49	1			1		
	20–100%	94	1.19	0.78–1.83	0.42	1.18	0.73–1.90	0.50
p-ERK1/2 (Thr202/Tyr204)	0%	61	1			1		
	10–100%	84	0.94	0.66–1.34	0.73	1.07	0.74–1.55	0.70
p-S6RP (Ser235/236)	0–10%	31	1			1		
	20–100%	111	1.19	0.82–1.71	0.36	1.11	0.76–1.62	0.59

Significance was defined as *p*-value ≤ 0.05

Proteins in tumour tissues were scored either for intensity of the staining (0–3) or percentage of positive tumour cells (0–100%)

*n* Number of patients, *HR* Hazard Ratio, *CI* confidence interval, *ECOG* Eastern Cooperative Oncology Group

^a^(Neo)adjuvant therapy could be endocrine therapy, chemotherapy or both

**Fig. 2:**
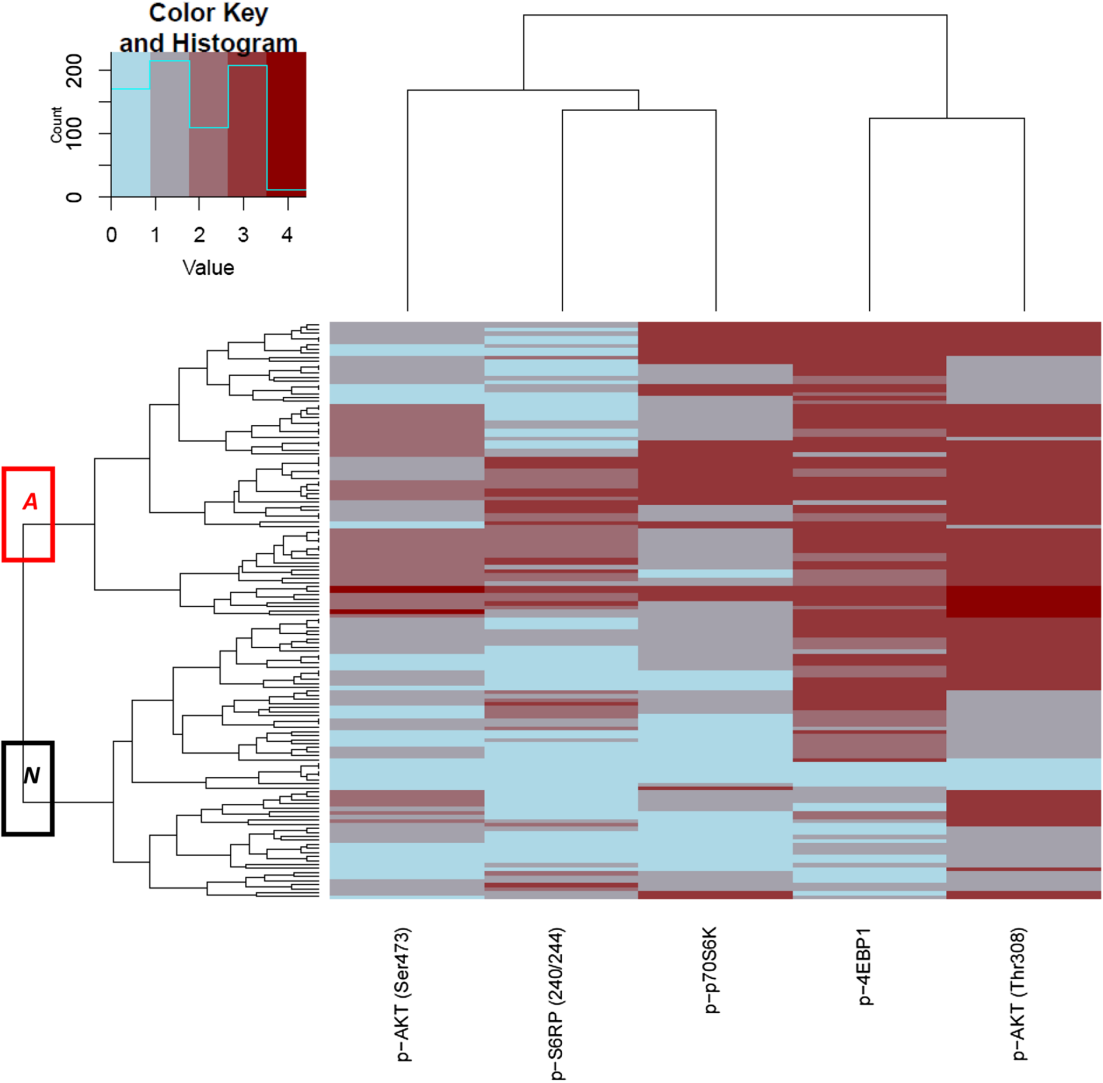
Unsupervised hierarchical clustering of continuous scores in primary tumour tissues of proteins associated with an activated PI3K signalling route visualised in a heatmap. The red box categorises patients in group *A* with a relatively activated pathway, while the black box categorises patients in group *N* with a relatively less-activated pathway

**Fig. 3:**
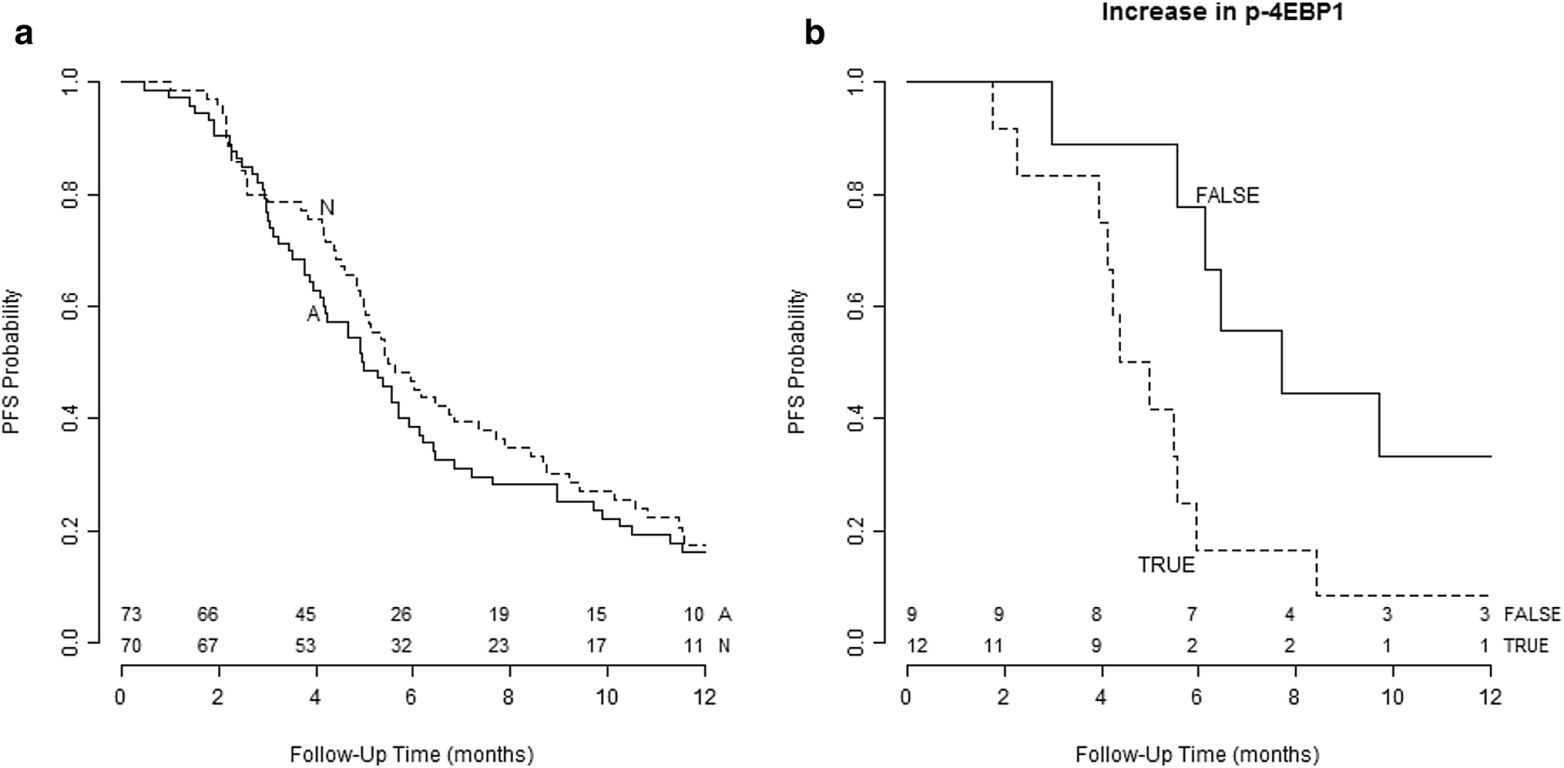
Kaplan**–**Meier curves representing progression-free survival (PFS) analyses according to **a** heatmap subgroup *A* with a preferentially activated PI3K pathway and *N* with a preferentially not activated PI3K pathway in primary tumour tissues showing no significant difference in PFS (multivariate HR 0.83, 95% CI 0.58–1.2, *p* = 0.34); **b** patients with (TRUE) and without (FALSE) upregulation of p-4EBP1 in pre-treatment biopsies compared to scorings in primary tumours showing significantly worse PFS in patients with upregulation of p-4EBP1 (multivariate HR 2.92, 95% CI 1.12–7.63, *p* = 0.029)

For a better presentation of an activated PI3K signalling route, we produced a heatmap of the 143 samples with scorings for all five proteins [p-AKT(Thr308), p-AKT(Ser473), p-4EBP1, p-p70S6K, p-S6RP(Ser240/244)]. Unsupervised hierarchical clustering resulted in subgroup (*A*) representing a preferentially activated PI3K pathway and a subgroup (*N*) with a relatively less-activated pathway (Fig. 2). Subgroup *A* contained preferentially more patients with PR-negative tumours compared to those in subgroup *N* (Table S3). PFS in patients allocated to *A* was not different from that in patients in *N* (univariate HR 0.81, *p* = 0.24; multivariate HR 0.83, *p* = 0.34) (Fig. 3a).

We then studied the effect of MAPK pathway activation by combining p-ERK1/2 and p-S6RP(Ser235/236). As expected, these proteins were significantly correlated (*p* = 0.00034). PFS was not different between patients with activation of the MAPK pathway (p-ERK1/2 positive and p-S6RP(Ser235/236) high) and those without pathway activation (univariate HR 1.15, *p* = 0.59; multivariate HR 1.2, *p* = 0.47).

To evaluate the effect of combined PI3K/MAPK pathway activation, we produced a heatmap with the continuous scores of seven proteins as described before (Kruger et al. 2018). There was no difference in PFS between subgroups with and without preferentially activated pathways (univariate HR 0.95, *p* = 0.79; multivariate HR 1.0, *p* = 0.99).

### Association of PFS with protein expression in pre-treatment biopsies

In 21 of 145 patients with staining results of their primary tumour tissue, a pre-treatment tumour biopsy was available. Characteristics of these 21 patients are shown in Table 1. None of the single PI3K/MAPK proteins correlated with PFS in both the uni- and multivariate analysis (Table S4).

Continuous staining scores of pre-treatment biopsies were compared with those in the corresponding primary tumours. Specifically, for p-4EBP1 and p-S6RP(Ser235/236), staining percentages were increased in pre-treatment biopsies (Table S5). Upregulation of p-4EBP1 was associated with a worse PFS on EVE/EXE (HR 2.91, 95% CI 1.12–7.63, *p* = 0.03), which remained significant in the multivariate analysis (HR 3.69, 95% CI 1.00–13.69, *p* = 0.05) (Table S5, Fig. 3b). Of interest, 17 of the 21 patients scored high p-4EBP1 levels in primary tumours of which nine biopsies of metastatic lesions showed p-4EBP1 upregulation. These nine patients still had a significantly worse PFS compared to the eight without upregulation (multivariate HR 10.0, *p* = 0.008). For p-S6RP(Ser235/236) neither univariate nor multivariate analysis showed an association with PFS (Table S5). Four of 20 pre-treatment tumour biopsies with PTEN scorings available showed downregulation, but this was not associated with PFS (data not shown).

## Discussion

Since the development of EVE/EXE for treatment of ER-positive, HER2-negative MBC patients refractory to an NSAI, several groups have tried to find markers that might predict which patients may have benefit from this combination or from everolimus plus endocrine therapy making use of IHC. IHC analysis of primary tumour tissues from 55 out of 111 patients who participated in the randomised phase II TAMRAD study (Treilleux et al. 2015) has shown that high levels of p-4EBP1 as well as low levels of p-AKT(Ser473) were associated with improved time-to-progression (TTP) on everolimus plus tamoxifen compared to that on tamoxifen alone. Okazaki et al. (Okazaki et al. 2018) have studied PTEN in primary tumour tissue of only 18 locally advanced BC or MBC patients on standard EVE/EXE, but found no relation with PFS. Although studies, including our attempt, on single PI3K-associated proteins in primary tumour tissue in the context of selecting MBC patients for EVE/EXE are few, it appears that this approach will not be of clinical value.

We performed unsupervised hierarchical clustering of five proteins related to PI3K pathway activation by which patients were divided into two subgroups with a preferentially activated (*A*) and preferentially not activated (*N*) pathway. When using a single marker as readout for total pathway activation, there is a risk to produce false positive or false negative results due to various feedback loops and cross-talks between canonical pathways. In our previous study in patients with primary ER-positive, HER2-negative primary breast cancer receiving adjuvant tamoxifen we have demonstrated that patients in the tumour subgroup with a preferentially activated PI3K and/or MAPK pathway derived no benefit from adjuvant tamoxifen, while those designated to the group without preferential activation had an improved relapse-free survival (Kruger et al. 2018). Here, hierarchical clustering analysis failed to identify patients who had a favourable outcome on EVE/EXE.

We tested two proteins associated with an activated MAPK pathway, p-ERK1/2 and p-S6RP(Ser235/236) in the context of possible benefit from treatment with EVE/EXE, but there was no relation with PFS. The group of Okazaki et al. (2018) have reported in only 18 ER-positive, HER2-negative MBC patients on EVE/EXE, that PFS was not significantly different between patients with high or low p-S6RP(Ser235/236) in primary tumour tissue. For the 55 patients randomised to everolimus plus tamoxifen or tamoxifen alone in the TAMRAD study, p-S6RP(Ser235/236) expression in primary tumour tissue was not associated with TTP (Treilleux et al. 2015). Of note, Hew et al. (2016) have described that MAPK activation predicts poor outcome in ER-positive high grade ovarian cancer and showed that MEK inhibition reversed anti-oestrogen resistance in an appropriate model system. Interestingly, our study cohort of ER-positive MBC patients contained more than 50% of patients with signs of an activated MAPK pathway in their primary tumour tissue, which suggests that the role of this pathway in endocrine resistance in breast cancer needs further study.

Use of actual tumour biopsies in clinical trials is being recommended to improve characterization of the pharmacodynamic effects of a drug, to understand the molecular processes involved in drug response or resistance, and to identify prognostic and predictive markers (Parseghian et al. 2017). In general, new tumour material is difficult to acquire, either because of refusal of the patient, the location is difficult to reach or because of inadequate sampling. Furthermore, an osseous lesion may not be useful for IHC of phosphorylated proteins. Despite these considerable hurdles, we could analyse 21 pre-treatment biopsies and showed that upregulation of p-4EBP1 as compared to staining results in primary tumour tissue was associated with a worse PFS on EVE/EXE. p-4EBP1 is considered to be a readout of an activated PI3K pathway and might indicate the presence of acquired endocrine resistance (Miller et al. 2011a; Osborne and Schiff 2011; Tryfonidis et al. 2016). This may explain the significant association between upregulation of p-4EBP1 and shorter PFS, since all our patients were pre-treated with at least an NSAI. A trend for a worse PFS in patients with high p-4EBP1 in primary tumour tissue was already observed in Figure S2D, suggesting that p-4EBP1 may be a prognostic marker. This is in line with a study from Karlsson et al. (2013) demonstrating a decreased distant recurrence-free survival in primary breast cancer patients unselected for subtype when p-4EBP1 was high in tumour tissue. Rojo et al. (2007) have described in unselected primary breast cancer patients that high p-4EBP1 was associated with the presence of lymph node metastases and a higher risk of locoregional recurrence. Due to the small number of patients and the lack of a control group in our study, no definite conclusions can be drawn whether p-4EBP1 or its upregulation is indeed associated with prognosis and whether it is useful as a marker for potential EVE/EXE benefit.

Apart from IHC, several groups have carried out mutational analysis of the *PI3K* pathway in an attempt to predict which ER-positive, HER2-negative MBC patients are candidates for EVE/EXE. In the BOLERO-2 study, *PIK3CA* mutation analysis in archival tumour samples of 302 patients has revealed that median PFS was longer for wild-type *PIK3CA* carriers in both treatment arms (Hortobagyi et al. 2016). PFS benefit from the combination appeared to be greater in those with exon-9 mutations than in those with exon-20 mutations. For the 45 primary tumour samples screened in the TAMRAD study, the number of patients with a *PIK3CA* mutation was too small for relevant statistical analysis (Treilleux et al. 2015). Among the 550 patients of the BOLERO-2 trial with plasma samples taken at the start of treatment to analyse circulating tumour (ct)DNA, median PFS of patients carrying wild-type or mutant *PIK3CA* was similar in the combination and the placebo arm (Moynahan et al. 2017). In the placebo arm, however, patients with an E545K/E542K mutation had a shorter PFS than those with wild-type *PIK3CA*. Yi et al. (Yi et al. 2019) have reported in a small study on pre-treatment ctDNA from 16 MBC patients receiving everolimus plus endocrine therapy that patients with the *PIK3CA*/H1047R mutation had a longer PFS than those with wild-type or other mutant forms of *PIK3CA.* Our ctDNA analyses including 10 relevant breast cancer genes recently performed in the current study cohort has revealed that patients with low/no ctDNA load or < 3 hotspot mutations experienced a longer PFS while treated with EVE/EXE (Kruger et al. 2020). Overall, mutational analysis of *PIK3CA* alone in MBC patients being candidates for EVE/EXE does not appear to have predictive value.

Our IHC study has limitations. There was no control group receiving exemestane plus placebo, since it was considered not ethical to include such a control group after the formal registration of EVE/EXE. It would be interesting if our p-4EBP1 findings in primary tissue could be validated in the BOLERO-2 study population to distinguish a prognostic or predictive effect. There are some challenges in IHC of phosphorylated proteins, because phospho-proteins can degrade in FFPE material (Siddiqui and Rimm 2010). However, our laboratory personnel has validated the staining procedure in tumour/non-tumour tissues with excellent results (Beelen et al. 2014; Kruger et al. 2018). Age of the tumour samples and different fixation procedures showed no effect of these variables on the phospho-protein expression levels (Beelen et al. 2014). We, therefore, believe that our data can be added to the existing information on potential markers for selecting patients for EVE/EXE.

In conclusion, we here demonstrate that IHC in primary tumour tissue of single proteins related to activated PI3K and/or MAPK pathways or hierarchical clustering of these proteins was not able to discriminate patients that might have benefit from standard EVE/EXE. Several proteins were upregulated in pre-treatment biopsies compared to primary tumour tissues of which p-4EBP1 was associated with a worse PFS. Further studies are necessary to assess the clinical significance of p-4EBP1 in ER-positive, HER2-negative breast cancer. Our study also shows the potential value of new biopsies to obtain insight in changes in tumour biology that affect prognosis and/or treatment choice.

## Electronic supplementary material

Below is the link to the electronic supplementary material.

Supplementary material 1 (DOCX 5470 kb)

## Data Availability

The data that support the findings of this study are available from the corresponding author upon reasonable request. Not applicable
