# Supplementary material for: PI3K pathway protein analyses in metastatic breast cancer patients receiving standard everolimus and exemestane
Source: J Cancer Res Clin Oncol. 2020 Jun 21;146(11):3013–23. doi: 10.1007/s00432-020-03291-x (PMC7519923; doi:10.1007/s00432-020-03291-x)
Supplement: Supplementary file 1 — Supplementary material 1 (DOCX 5470 kb) [file 432_2020_3291_MOESM1_ESM.docx]

# PI3K pathway protein analyses in metastatic breast cancer patients receiving standard everolimus and exemestane

Dinja T Kruger, Mark Opdam, Vincent van der Noort, Joyce Sanders, Michiel Nieuwenhuis, Bart de Valk, Karin J Beelen, Sabine C Linn, Epie Boven

## Supplementary Materials and methods

**Study design**

Patients were eligible when they were ≥18 years old postmenopausal women with ER-positive, HER2-negative metastatic breast cancer. ER-positive disease was defined as ≥10% of tumour cells showing nuclear staining, according to Dutch pathological guidelines. Patients needed to be refractory to an NSAI, defined as a recurrence ≤12 months of adjuvant anastrozole or letrozole or having progressed while on or within one month of discontinuing non-steroidal aromatase inhibitor (NSAI) treatment for metastatic disease, but the NSAI did not have to be the last systemic treatment prior to enrolment. Planned next treatment had to be standard everolimus plus exemestane. Previous treatment with mTOR inhibitors was not allowed. Patients were excluded when they received hormone replacement therapy, or those (sero)positive for HIV, hepatitis B or C or with inadequate bone marrow, liver or renal function.

## Supplementary Tables

### Table S1. Participating hospitals and principal investigators of the Everolimus Biomarker Study

| **Participating hospital** | **Principal investigator** |
| --- | --- |
| Amphia Ziekenhuis | Dr. J.B. Heijns |
| Antoni van Leeuwenhoek - NKI | Prof. Dr. S.C. Linn |
| BovenIJ Ziekenhuis | Dr. S.E. Dohmen |
| Bravis locatie Roosendaal en Bergen op Zoom | Drs. H. Droogendijk |
| Canisius Ziekenhuis | Dr. C.M.P.W. Mandigers |
| Deventer Ziekenhuis | Drs. L.W. Kessels |
| Elisabeth-TweeSteden Ziekenhuis | Dr. J.M.G.H. van Riel |
| Erasmus Universitair MC | Dr. A. Jager |
| Flevoziekenhuis | Dr. D.W. Sommeijer |
| Gelre Ziekenhuizen | Dr. J. Oulad Hadj |
| Groene Hart Ziekenhuis | Dr. B. Tanis |
| HagaZiekenhuis | Drs. Houtsma and Dr. J.P. Portielje |
| Ikazia Ziekenhuis | Dr. F.E. de Jongh |
| Isala Klinieken | Dr. A.H. Honkoop |
| Leids Universitair MC | Dr. J.R. Kroep |
| MC Haaglanden en Bronovo-Nebo | Drs. H.M. Oosterkamp |
| MC Leeuwarden | Dr. H. de Graaf |
| Maxima Medisch Centrum | Dr. M.W. Dercksen |
| NW Ziekenhuisgroep, Locatie Den Helder | Dr. J.C. Berends |
| Reinier de Graaf Groep | Dr. K. Beelen and Dr. M.M.E.M. Bos |
| Rijnstate Ziekenhuis Arnhem | Drs. M.J.D.L. van der Vorst |
| Spaarne Ziekenhuis | Dr. B. de Valk |
| Tergooi ziekenhuizen locatie Hilversum | Dr. S.A. Luykx |
| UMC Utrecht | Drs. R.M. Bijlsma |
| Viecuri Medisch Centrum | Dr. A.J. van de Wouw |
| Vlietland Ziekenhuis | Drs. Q.C. van Rossum |
| VUmc | Dr. I.R.H.M. Konings and prof. dr. E. Boven |
| Zuyderland, locatie Orbis en Atrium MC | Dr. F.L.G. Erdkamp |

### Table S2. Antibodies used for immunohistochemistry, scoring procedures and kappa coefficients to determine the interobserver variability

| Antibody | Art. No. (clone)^a^ | Scoring system | Cut-off for binary score | Kappa for binary score | Number of cores used for kappa calculation |
| --- | --- | --- | --- | --- | --- |
| PTEN | 9559 (138G6) | cytoplasmic intensity (0-3) | 0 *vs* 1-3 | 0.75 | 126 |
| p-AKT(Thr308) | 2965 (C31E5E) | cytoplasmic intensity (0-3) | 0 *vs* 1-3 | 0.53 | 133 |
| p-AKT(Ser473) | 4060 (D9E) | cytoplasmic intensity (0-3) | 0-1 *vs* 2-3 | 0.70 | 94 |
| p-4EBP1(Ser65) | 9456 (174A9) | percentage of tumour cells with nuclear staining (0% - 100%) | 0-50 *vs* 51-100 | 0.59 | 112 |
| p-p70S6K (Thr389) | 9206 (1A5) | cytoplasmic intensity (0-3) | 0 *vs* 1-3 | 0.44 | 97 |
| p-S6RP (Ser240/244) | 5364 (D68F8) | percentage of tumour cells with cytoplasmic and membranous staining (0% - 100%) | 0-19 *vs* 20-100 | 0.93 | 54 |
| p-ERK1/2 (Thr202/Tyr204) | 4370 (D13.14.4E) | percentage of tumour cells with nuclear staining (0% - 100%) | 0 *vs* 1-100 | 0.85 | 69 |
| p-S6RP (Ser235/236) | 2211 | percentage of tumour cells with cytoplasmic and membranous staining (0% - 100%) | 0-19 *vs* 20-100 | 0.55 | 85 |

^a^all from Cell Signaling Technology, Danvers, MA, US

Ref: Beelen et al, *Breast Cancer Res*, 16:R6, 2014; Kruger et al, *Brit J Cancer*, 119, 832–839, 2018

### Table S3. Association between clinico-pathological characteristics at study baseline and heatmap subgroups *A* and *N* classified by unsupervised hierarchical clustering of continuous scorings of phosphorylated PI3K-related proteins in primary tumour tissues

|  |  | Heatmap subgroups (n = 143)^a^ | | |
| --- | --- | --- | --- | --- |
|  |  | *A* | *N* |  |
|  |  | n (%) | n (%) | *p*-value^b^ |
| Age | <65 years | 43 (30) | 36 (25) | 0.4 |
|  | ≥65 years | 30 (21) | 34 (24) |  |
| ECOG status at screening visit | 0 | 33 (23) | 24 (17) | 0.23 |
|  | 1-2 | 40 (28) | 46 (32) |  |
| Disease-free interval | <12 months | 18 (13) | 13 (9) | 0.42 |
|  | ≥12 months | 55 (38) | 57 (40) |  |
| Progesterone receptor status | Negative | 23 (16) | 8 (6) | **0.007** |
| of the primary tumour^c^ | Positive | 49 (34) | 58 (41) |  |
| (Neo)adjuvant therapy^d^ | No | 32 (22) | 30 (21) | 0.38 |
|  | Yes | 41 (29) | 40 (28) |  |
| Bone only disease | No | 60 (42) | 58 (41) | 1 |
|  | Yes | 13 (9) | 12 (8) |  |
| Palliative chemotherapy | No | 54 (38) | 48 (34) | 0.58 |
|  | Yes | 19 (13) | 22 (15) |  |
| T-stage^e^ | T1-2 | 44 (31) | 48 (34) | 0.55 |
|  | T3-4 | 18 (13) | 15 (10) |  |

^a^ proteins used in the unsupervised hierarchical clustering analysis to generate the heatmap were

p-AKT(Thr308), p-AKT(Ser473), p-4EBP1(Ser65), p-p70S6K(Thr389) and p-S6RP(Ser240/244)

^b^ Fisher’s exact test based on cases without missing values

^c^ five patients had missing PR status

^d^ (Neo)adjuvant therapy could be endocrine therapy, chemotherapy or both

^e^ T-stage was unknown of 10 patients in group *A* and seven in group *N*

Abbreviations: *A*: patients in heatmap subgroup *A* with a more activated PI3K pathway

in primary tumour tissue; *N*: patients in heatmap subgroup *N* with a less activated PI3K

pathway; n: number of patients, ECOG: Eastern Cooperative Oncology Group

### Table S4. Uni- and multivariate Cox analyses of scoring of single proteins in pre-treatment biopsies and progression-free survival

|  |  | Progression-free survival | | | | | | |
| --- | --- | --- | --- | --- | --- | --- | --- | --- |
|  |  |  |  | Univariate analysis |  |  | Multivariate analysis |  |
|  |  | n | HR | 95% CI | p-value | HR | 95% CI | *p*-value |
| PTEN^a^ | 0 | 2 | 1 |  |  |  |  |  |
|  | 1-3 | 19 | 0.69 | 0.15 - 3.10 | 0.63 | 0.82 | 0.17 - 3.97 | 0.81 |
| p-AKT (Thr308) | 0 | 0 | 1 |  |  |  |  |  |
|  | 1-3 | 21 | NA | NA | NA | NA | NA | NA |
| p-AKT (Ser473) | 0-1 | 11 | 1 |  |  |  |  |  |
|  | 2-3 | 10 | 0.94 | 0.37 - 2.38 | 0.90 | 1.23 | 0.38 - 3.97 | 0.73 |
| p-4EBP1 (Ser65) | 0-50% | 3 | 1 |  |  |  |  |  |
|  | 51-100% | 18 | 3.49 | 0.80 - 15.33 | 0.10 | 5.03 | 0.64 - 39.66 | 0.13 |
| p-p70S6K (Thr389) | 0 | 2 | 1 |  |  |  |  |  |
|  | 1-3 | 19 | 0.56 | 0.13 - 2.53 | 0.45 | 0.61 | 0.13 - 2.88 | 0.54 |
| p-ERK1/2 (Thr202/Tyr204) | 0% | 3 | 1 |  |  |  |  |  |
|  | 1 – 100% | 18 | 0.97 | 0.27 - 3.49 | 0.97 | 1.06 | 0.13 - 8.61 | 0.96 |
| p-S6RP (Ser235/236) | 0 – 10% | 1 | 1 |  |  |  |  |  |
|  | 20 – 100% | 20 | 1.06 | 0.14 - 8.14 | 0.96 | 1.26 | 0.16 - 10.17 | 0.83 |

^a^PTEN negative = 0, PTEN positive = 1-3

Abbreviations: n: number of patients; HR: Hazard Ratio; CI: Confidence Interval

### Table S5. Uni- and multivariate Cox analyses of pre-treatment biopsies without (No) upregulation *vs* those with (Yes) upregulation of proteins compared to continuous staining scores in primary tumour tissues and progression-free survival

|  |  | Progression-free survival | | | | | | |
| --- | --- | --- | --- | --- | --- | --- | --- | --- |
|  |  |  |  | Univariate analysis |  |  | Multivariate analysis |  |
|  |  | n | HR | 95% CI | p-value | HR | 95% CI | *p*-value |
| p-AKT(Thr308) | No | 12 | 1 |  |  |  |  |  |
|  | Yes | 8 | 0.77 | 0.30 - 1.97 | 0.58 | 0.79 | 0.27 - 2.34 | 0.67 |
| p-AKT(Ser473) | No | 11 | 1 |  |  |  |  |  |
|  | Yes | 10 | 0.93 | 0.38 - 2.25 | 0.87 | 1.06 | 0.36 - 3.12 | 0.91 |
| p-4EBP1(Ser65) | No | 9 | 1 |  |  |  |  |  |
|  | Yes | 12 | 2.91 | 1.12 - 7.63 | **0.03** | 3.69 | 1.0 - 13.69 | **0.05** |
| p-p70S6K(Thr389) | No | 15 | 1 |  |  |  |  |  |
|  | Yes | 6 | 0.86 | 0.31 - 2.40 | 0.78 | 1.05 | 0.31 - 3.53 | 0.94 |
| p-ERK1/2 | No | 12 | 1 |  |  |  |  |  |
| (Thr202/Tyr204) | Yes | 9 | 0.69 | 0.28 - 1.71 | 0.42 | 0.61 | 0.21 - 1.75 | 0.36 |
| p-S6RP | No | 6 | 1 |  |  |  |  |  |
| (Ser235/236) | Yes | 14 | 1.62 | 0.60 - 4.38 | 0.34 | 3.39 | 0.93 - 12.34 | 0.06 |

Abbreviations: n: number of patients; HR: Hazard Ratio; CI: Confidence Interval

## Supplementary Figures

### Figure S1 Participants in the Everolimus Biomarker Study

Included patients

N= 175

Potentially eligible patients

N= 178

Patients included in the IHC analyses

N= 145

Patients excluded:

- Discontinued both drugs within one month due to toxicity (N = 4)
- No tumor tissue available (N = 26)

Patients excluded:

- HER2-positive disease in extra biopsy (N = 1)
- Serum aspartate aminotransferase (AST) > 5 ULN and total serum bilirubin >1.5 ULN at the start of the treatment (N = 1)
- Never started treatment with everolimus plus exemestane (N = 1)

###
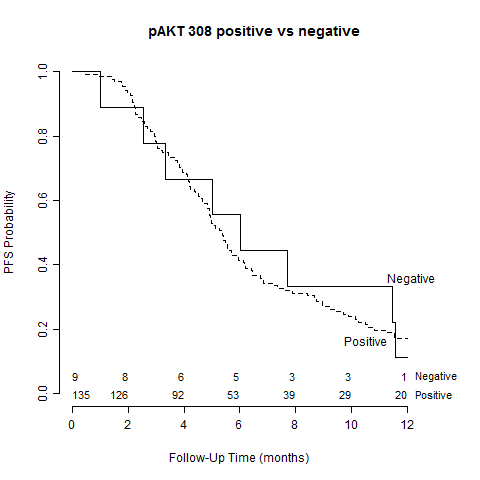
Figure S2. Kaplan-Meier curves of binary scores of single proteins in primary tumour tissues and progression-free survival


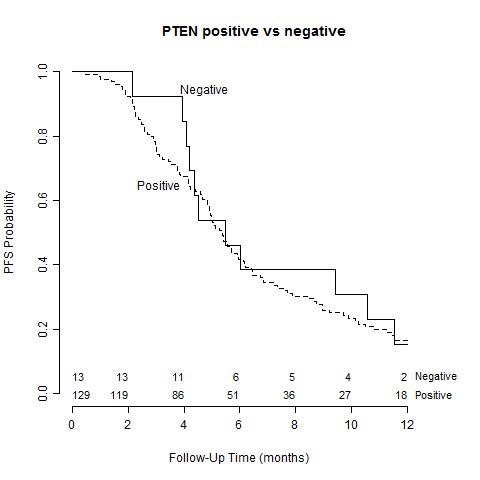


**B**

**A**


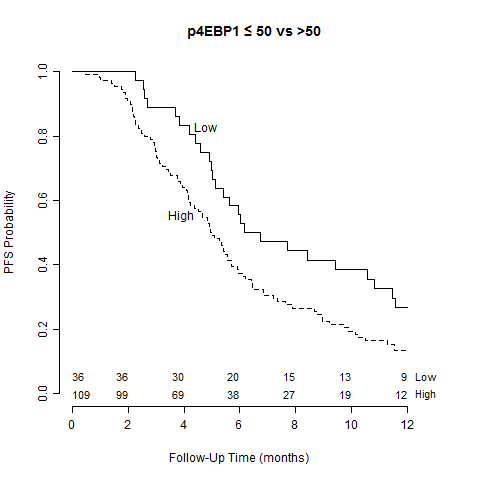

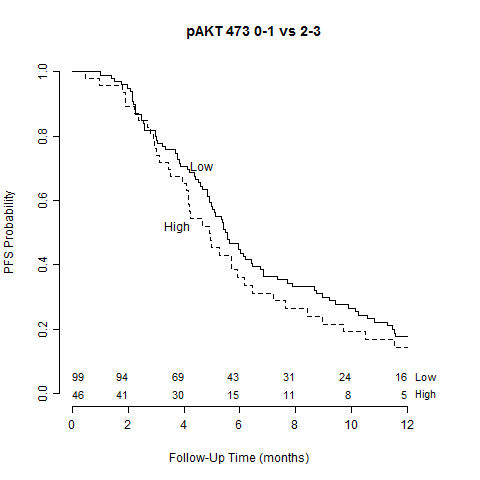


**C**

**D**


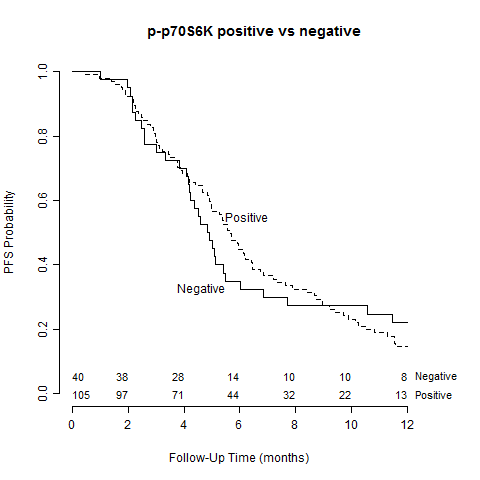
**Figure S2** **continued**.


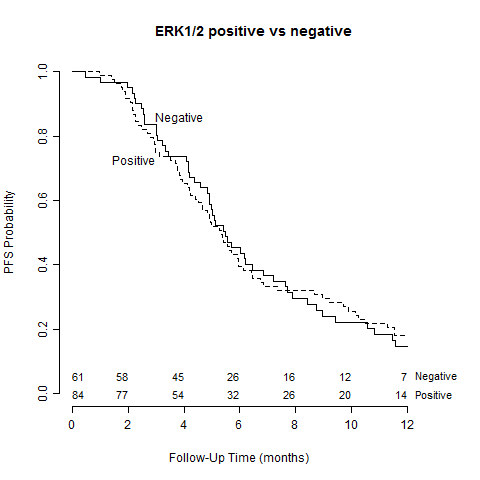
**
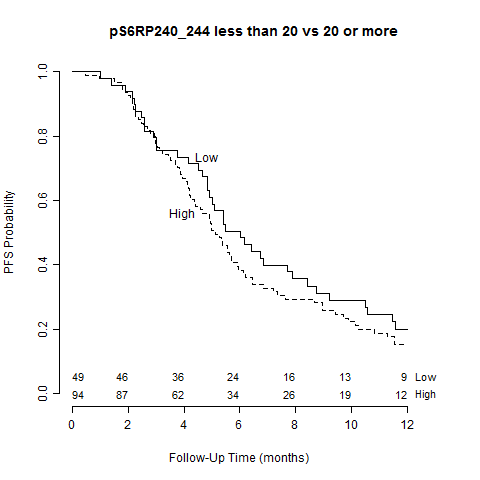

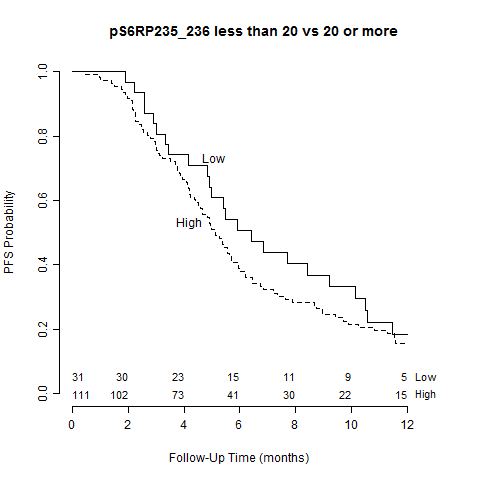
**

**F**

**E**

**G**

**H**

Kaplan-Meier curves showing progression-free survival (PFS) for **A**. PTEN; **B**. p-AKT(Thr308); **C**. p-AKT(Ser473); **D**. p-4EBP1; **E**. p-p70S6K; **F**. p-ERK1/2; **G**. p-S6RP(Ser235/236) and **H**. p-S6RP(Ser240/244)
